# Association between nutritional risk status and health related quality of life: An investigation on the aging patients with cardiovascular disease

**DOI:** 10.34172/jcvtr.2023.32903

**Published:** 2023-12-30

**Authors:** Leili Faraji Gavgani, Somayeh Alipour, Roghayeh Khabiri, Delara Laghousi, Parvin Sarbakhsh, Haniyeh Farajiazad, Mahdieh Abbasalizad Farhangi, Leila Jahangiry

**Affiliations:** ^1^Department of Statistics and Epidemiology, Faculty of Health, Tabriz University of Medical Sciences, Tabriz, Iran; ^2^Health Education and Health Promotion Department, School of Health, Tabriz University of Medical Sciences, Tabriz, Iran; ^3^Tabriz Health Services Management Research Center, Tabriz University of Medical Sciences, Tabriz, Iran; ^4^Social Determinants of Health Research Centre, Health Management and Safety Promotion Research Institute, Tabriz University of Medical Sciences, Tabriz, Iran; ^5^Madani Heart Center, Cardiovascular Research Center, Tabriz University of Medical Sciences, Tabriz, Iran; ^6^Department of Community Nutrition, Faculty of Nutrition, Tabriz University of Medical Sciences, Tabriz, Iran

**Keywords:** Undernutrition, Nutrition risk screening, Elderly, Heart disease

## Abstract

**Introduction:**

Acknowledging the considerable influence of undernutrition on health outcomes and HRQOL, this study sought to appraise the nutritional risk status of elderly patients with cardiovascular diseases (CVD) through the utilization of the Nutritional Risk Screening (NRS). Additionally, the investigation aimed to evaluate the correlation between NRS status and HRQOL within the context of patients referred to a cardiac hospital in Tabriz, Iran.

**Methods:**

This cross-sectional study was conducted in Tabriz, Iran. The participants were selected randomly from patients referring to Shahid Madani Heart Hospital, a comprehensive university hospital during July to December 2018. A linear regression was used for control of confounding variables (age, gender, education level, marital status, and income levels) and predict the relationship between nutrition risk status and HQRL.

**Results:**

Of the 200 patients with CVD participated in this study, 68 (34%) of participants had normal nutrition status, 108 (54%) were at risk for undernutrition, and 24 (12%) had undernutrition. A total of 24 aging patients with undernutrition, 13 (54%) were divorced or widowed. 86% of patients with diabetes were at risk for undernutrition and 13.9% had undernutrition. There were statistically significant relationship between undernutrition and HRQOL dimensions, age, gender, and marital status.

**Conclusion:**

The study revealed a correlation between elevated undernutrition scores in patients and factors such as older age, female gender, and marital status of being divorced or widowed. Furthermore, the results imply that a notable elevation in the risk score for undernutrition in patients is significantly linked to impaired HRQOL among elderly individuals with CVD.

## Introduction

 Nutritional status among older adults is an often overlooked concern in developing countries, despite its significant impact on diseases, disability, quality of life, and mortality rates among the elderly worldwide.^1^ Older population are particularly vulnerable to undernutrition due to various physiological, economic, and social risk factors that detrimentally affect their physical and mental health.^2^ Aging is commonly accompanied by reduced appetite, weight loss, and the onset of acute and chronic diseases, all of which contribute to declines nutritional status.^3^ Furthermore age-related declines in taste and smell perception, dental health, and stomach acid production can lead to decreased food intake, diminished body mass, and reduced nutrient reserves.^4,5^ Undernutrition a prevalent risk factor for clinical disorders, often goes undiagnosed in its early stages; resulting in untreated concequences.^5-8^

 Identifying undernutrition among the elderly can be challenging, making early detection and the development of effective prevention programs crucial for the aging population especially for patients with cardiovascular diseases (CVD).^9,10^ The prevalence of undernutrition in older populations worldwide varies from 10% to 85%, influenced by the diverse methods used for nutritional risk screening.^11,12^

 Health-related quality of life (HRQOL) is a comprehensive concept that encompasses self-reported physical and mental health status of patients.^13^ HRQOL is a sensitive indicator for assessing the consequences of diseases and outcomes of interventions among patients.^14^

 Acknowledging the considerable influence of undernutrition on health outcomes and HRQOL, this study sought to appraise the nutritional risk status of elderly patients with CVD through the utilization of the Nutritional Risk Screening (NRS). Additionally, the investigation aimed to evaluate the correlation between NRS status and HRQOL within the context of patients referred to a cardiac hospital in Tabriz, Iran.

## Materials and Methods

###  Study design and participants

 This cross-sectional study was conducted in Tabriz, Iran. The participants were selected randomly from patients referring to Shahid Madani Heart Hospital, a comprehensive university hospital within Tabriz University of Medical sciences during July to December 2018. Specifically, patients aged 60 years and older who had recently been admitted the hospital were included in the study. The written informed consent was obtained from the patients who agreed to participate in the study.

###  Sample size

 The study aimed to estimate the proportion in the society, using a sample size formula for estimating proportions in the community with a specified confidence level and precision (P = 50%, 7% accuracy, and 95% confidence interval). Considering an unlimited population size, the estimated required sample size was 196 individuals. To account for potential dropouts, the final sample size was set at 200 patients.

###  Inclusion criteria

 Participants were included in the study if they met the following criteria: 1) Patients aged 60 years and over, 2) They were hospitalized, 3) They provided personal consent to participate in the study. Exclusion criteria were having cognitive disorders and no interest to fulfill the study questionnaires.

###  Measurements 

 To assess the nutritional status of the patients, the NRS-2002 tool was employed. NRS-2002 screens the risk of undernutrition in hospitalized adult patients. The validity and reliability of the NRS questionnaire had previously been established.^15^ The tool comprises four items, including Body Mass Index (BMI) < 20.5 kg/m^2^, weight loss in the last three months, reduced dietary intake in the last week, and the presence of a serious illness. The total NRS score ranges from 0 to 3, with a score lower than 3 indicating no risk of undernutrition, and a score of 3 or higher indicating a high risk or clear undernutrition.^16^ LEIPAD instrument was used to assess quality of life and evaluate long-term and acute care situations among patients. Validity and reliability of the LEIPAD questionnaire was approved among the Iranian elderly population.^17^ Blood samples were collected for the measurement of total cholesterol, triglycerides (TGs), low-density lipoprotein (LDL) cholesterol, high density lipoprotein cholesterol (HDL-C), and fasting blood sugar (FBS). Fasting for 12–14 hours was required before blood sampling. Anthropometric parameters such as height, weight, waist circumference, arm circumference, hip circumference, and leg circumference were measured and recorded. Socioeconomic status was assessed by dividing the number of family members in the household by the square meter of the home.^18^ Randomly selected patients completed the study questionnaire within 24 hours of admission. The study received approval from the Ethics Committee for Medical Research.

###  Statistical analysis

 Descriptive statistics, including mean and standard deviation (SD) for quantitative variables and frequency and percentage for categorical variables were employed. The Chi-Square and Fisher’s exact tests were used for comparing qualitative variables, and ANOVA was used for comparing quantitative variables among three groups: no undernutrition, risk for undernutrition, and undernutrition patients. A linear regression was used for control of confounding variables and predict the relationship between nutrition risk status and HQRL. The main variables (undernutrition as independent variable and HQRL dimensions as dependent variables) with all confounders (age, gender, education level, marital status, and income level) were added to the model and the final set of covariates was selected using backward limitation approach. Statistical analyses were performed using the Statistical Package for Social Science, Version 18, for Windows (SPSS Inc., Chicago, IL, USA). In all tests, a value of p < 0.05 was considered statistically significant.

## Results

 Out of the 200 patients with (CVD) included in the study, 68 (34%) had normal nutrition status, 108 (54%) were at risk for undernutrition, and 24 (12%) had undernutrition. The patients with undernutrition were significantly older than the other patients (P < 0.0001). A higher proportion of women were at risk for undernutriotion or had undernutrition compared to men (Table 1).

**Table 1:** Demographic characteristics of the patients (n = 200)

**Variable**	**No undernutrition (NRS score=0) (n=68)**	**Risk for undernutrition (NRS score=1-2) (n=108)**	**undernutrition (NRS score=3-5) ** **(n=24)**	***P*** ** value**
Age (yrs) mean (SD)	64.5 (3.1)	69.9 (7.5)	71.3 (6.9)	< 0.001
Gender n (%)				< 0.001*
Men	46 (41.4)	58 (52.3)	7 (6.3)	
Women	22 (24.7)	50 (56.2)	17 (19.1)	
Marital status n (%)				0.001*
Married	58 (40.8)	73 (51.4)	11 (7.7)	
widowed /divorced	10 (17.2)	35 (60.3)	13 (22.4)	
Employment status n (%)				0.001*
employed	19 (55.9)	14 (41.2)	1 (2.9)	
Retired	20 (46.5)	21 (48.8)	2 (4.7)	
Unemployed	27 (23.3)	68 (58.6)	21 (18.1)	
Education level n (%)				0.275
Less than diploma	61 (33.5)	97 (53.3)	24 (13.2)	
Diploma and above	7 (38.9)	11 (61.1)	0	
Smoking n (%)				
Yes	12 (44.4)	14 (51.9)	1 (3.7)	0.279*
Social economic status n (%)				0.03
< median	32 (32.7)	51 (52.0)	15 (15.3)	
≥ median	36 (35.3)	57 (55.9)	9 (8.8)	

*Pearson Chi-Square **Fisher’s Exact Test *P*<0.05 is statistically significant.

 The history of diseases among patients with normal nutrition, at risk for undernutrition and with undernutrition was presented in Table 2. A significant association was observed between the patients’ history of diseases and their nutritional status.

**Table 2:** Comparison of diseases characteristic among CVD patients without malnutrition, at risk for malnutrition, and with malnutrition (n = 200)

	**No undernutrition (NRS score=0) (n=68) n (%)**	**Risk for under nutrition (NRS score=1-2) (n=108) n (%)**	**undernutrition (NRS score=3-5) ** **(n=24),n (%)**	***P*** ** value**
History of heart attack Yes	27 (36.5)	39 (52.7)	8 (10.8)	0.05
History of heart disease Yes	48 (31.8)	82 (54.3)	21 (13.9)	0.05
History of diabetes Yes	0 (0.0)	62 (86.1)	10 (13.9)	< 0.001
History of hyperlipidemia Yes	14 (18.2)	49 (63.6)	14 (18.2)	< 0.001
History of high blood pressure (Yes)	42 (33.9)	66 (53.2)	16 (12.9)	0.382
History of cardiac surgery (Yes)	40 (32.5)	69 (56.1)	14 (11.4)	0.515
Comorbidity Yes	16 (22.5)	43 (60.6)	12 (16.9)	0.025
History of stroke Yes	68 (34.2)	108 (54.3)	23 (11.6)	0.12

^*^ Pearson Chi-Square ^**^ Fisher’s Exact Test *P*<0.05 is statistically significant.

 Clinical and anthropometric parameters of CVD patients in the three groups of patients were shown in Table 3. FBS and TGs were significantly higher among patients with undernutrition status than other patients. Additionally, among patients with undernutrition, weight, BMI, waist and leg circumference were lower than other patients. Although systolic blood pressure among patients with normal nutrition status was lower than other patients, it was not statistically significant. Additionally, patients with undernutrition or at risk for undernutrition had significantly impaired HRQOL (Table 4).

**Table 3:** Clinical and anthropometric characteristics of older adults with and without malnutrition with cardiovascular disease (n = 200)

**Variable**	**No undernutrition (NRS score=0) (n=68) n (%)**	**Risk for undernutrition (NRS score=1-2) (n=108) n (%)**	**undernutrition (NRS score=3-5) ** **(n=24) n (%)**	***P*** value*
Systolic BP (mmHg)	125.5 (19.9)	131.1 (24.2)	130.0 (18.2)	0.275
Diastolic BP (mmHg)	72.0 (14.9)	71.9 (14.9)	72.0 (14.7)	0.999
Cholesterol (mg/dl)	147.5 (46.1)	151.5 (43.7)	165.6 (42.2)	0.220
FBS (mg/dl)	119.4 (3.5)	151.6 (67.3)	165.6 (42.2)	0.003
LDL-chol (mg/dl)	100.4 (43.5)	97.3 (41.5)	118.4 (46.9)	0.151
HDL-chol (mg/dl) Men women	30 (8.4) 40.4 (28.8)	32.3 (10.8) 34.8 (12.2)	37.4 (7.7) 34.8 (8.3)	0.141 0.42
TG (mg/dl)	108.5 (41.6)	137.5 (68.1)	122.2 (47.8)	0.006
Weight (Kg)	83.8 (16.7)	77.8 (13.1)	65.3 (14.4)	< 0.0001
Waist circumference (cm)	94.3 (10.4)	92.5 (11.1)	86.2 (9.2)	0.007
Leg circumference (cm)	23.7 (2.6)	23.4 (2.3)	21.6 (5.0)	0.009
BMI (kg/m^2^)	27.9 (3.4)	27.3 (4.2)	23.3 (3.8)	< 0.0001

*Note:* BP: blood pressure, FBS: Fasting blood sugar, LDL: Low-density lipoprotein, HDL: High density lipoprotein cholesterol, TG: triglycerides, BMI: Body Mass Index, *Independent Sample T-test *P*<0.05 is statistically significant.

**Table 4:** The relationship between nutritional risk screening score and health related quality of life among older adults with CVD (n = 200)

	**Range of scores **	**No undernutrition (NRS score=0) (n=68)**	**Risk for undernutrition (NRS score=1-2) (n=108)**	**undernutrition (NRS score=3-5) ** **(n=24)**	***P*** ** value**
Physical Functioning	0-15	9.4 (2.7)	6.7 (2.9)	7.0 (2.9)	< 0.0001
Self-care	0-18	16.7 (2.4)	13.4 (4.3)	12.3 (5.1)	< 0.0001
Depression and anxiety	0-12	8.3 (2.6)	7.3 (2.9)	6.1 (3.0)	0.006
Cognitive Functioning	0-15	10.8 (2.2)	8.6 (2.9)	8.4 (2.7)	< 0.0001
Social Functioning	0-9	4.4 (1.8)	3.9 (1.7)	4.1 (1.7)	0.297
Sexual Functioning	0-6	2.6 (1.2)	1.5 (1.2)	1.2 (1.2)	< 0.0001
Life Satisfaction	0-18	9.4 (3.8)	8.0 (3.8)	9.4 (3.1)	0.031

*P*<0.05 is statistically significant.

 The relationship between nutritional risk status and HRQOL with available covariates (age, gender, and marital status) are shown in Table 5. The table provides insights into how different factors related to the risk of undernutrition are associated with various aspects of the HRQOL, as indicated by the coefficients and *P* values from linear regression analyses.

**Table 5:** Linear regression between the quality of life and risks for undernutrition

	**Risk of under- nutrition **	**Age **	**Gender**	**Marital status **
	**B;** ***P***** value**			
Physical Functioning	-0.455; 0.041	-0.203; < 0.0001	-	-
Self-care	-0.462; 0.073	-0.407; < 0.0001	-0.870; 0.049	-
Depression and anxiety	-0.508; 0.030	-0.085; 0.005	-0.95; 0.018	-
Cognitive Functioning	-0.483; 0.024	-0.158; < 0.0001	-	-
Social Functioning	0.045; 0.756	-	-	-
Sexual Functioning	-0.182; 0.040	-0.080; < 0.0001	-0.45; 0.006	0.556; 0.004
Life Satisfaction	0.014; 0.966	-	-	-

*P*<0.05 is statistically significant.

## Discussion

 This study aimed to investigate the nutritional risk status and its correlation with CVD risk factors and HRQOL in the elderly patients aged 60 years and above who were admitted to a comprehensive heart hospital in Tabriz. Our results revealed that 12% of patients with CVD were identified as being undernutrished, 54% at risk for undernutrition, and 34% had normal nutritional status. These findings align with a study by Aliabadi et al where a prevalence of 12% undernutrition among older individuals was using Mini Nutritional Assessment (MNA) tool.^19^ Several other studies from different countries have also found similar rates of undernutrition among elderly patients.^20-23^ For example, a study in Nepal in 2021 reported 11.6% with undernutrition and 49.7% at risk of undernutrition.^21^ However, the prevalence of undernutrition among hospitalized elderly patients in Egypt in 2013 was 18%,^22^ and in Lebanon in 2014, it was 6.1%.^23^ These discrepancies are likely linked to variations in the types of diseases and hospitalization conditions.

 This study specifically focused on newly admitted patients with CVD in a heart hospital. Substantial evidence supports the notion that undernutrition can impact the prognosis of various diseases, including CVD.^24-26^ A study by Arikawa et al,^26^ which used the geriatric nutritional risk index (GNRI) to assess undernutrition, demonstrated that patients with undernutrition had a higher risk of major cardiovascular and cerebrovascular events compared to those without undernutrition. BMI is a significant risk factor in both the NRS scale and GNRI for evaluating nutritional status. However, GNRI combines BMI and serum albumin levels to predict nutritional status among older adults.^27^ Undernutrition can have adverse consequences on CVD,^28,29^ and while it is influenced by multiple risk factors, inflammation plays a central role in its development. Chronic inflammation can lead to CVD by increasing oxidative stress and causing severe endothelial dysfunction.^30^ Additionally, patients with undernutrition and CVD may require intensive care to prevent severe cardiovascular events. Management of nutritional status, feeding, and energy intake may also enhance the prognosis for these patients.

 Our findings indicated that women with CVD were significantly more at risk of undernutrition than men. A study in Iran in 2013 revealed that diet-related risk factors for CVD were more prevalent in women than men.^31^ Women obtained a higher portion of their energy intake from fats. It is possible that women are trying to maintain a lower-calorie diet while increasing physical activity and adopting dietary patterns to reduce CVD risk factors.^31^ Moreover, evidence suggests that women have more comorbid conditions and general risk factors for CVD compared to men. Women with CVD were more likely to have obesity, diabetes mellitus, hypercholesterolemia, and hypertension,^32,33^ which could explain the observed higher risk of undernutrition among women in this study with potentially severe consequences.

 Furthermore, our results showed significant age differences between elderly patients with and without undernutrition, with undernourished patients being notably older. Similar findings were observed by Alzahrani et al^33^ who attributed undernutrition in older patients to decreased food intake, loss of appetite, digestive problems, and swallowing difficulties.

 In our study, patients with undernutrition had significantly higher levels of fasting blood sugar (FBS) and triglycerides (TG) compared to other patients. Additionally, these undernourished patients had significantly lower weight, BMI, waist and leg circumference.^34^ Patients at risk of or with undernutrition also had significantly impaired HRQOL compared to those with normal nutrition. These results align with other studies involving CVD patients, underscoring the multifaceted impact of nutritional status on HRQOL.^35,36^

 To further enhance our understanding of this issue, additional research is needed to monitor both the quality and quantity of dietary intake. Furthermore, the need for nutritional support, dietary planning using a treatment approach, and specific interventions during hospitalization and procedures should be explored, particularly given the effects of invasive treatments on nutritional status.

 The study’s exclusive focus on patients referred to a heart hospital in Tabriz, Iran, may limit the generalizability of the findings to a broader population. Results may not accurately reflect the nutritional risk status and its impact on HRQOL in diverse healthcare settings. Additionally, the cross-sectional nature of the study provides a snapshot of the relationship between nutritional risk status and HRQOL at a specific point in time. Longitudinal studies would be needed to establish causality and assess changes over time.

## Conclusion

 The study revealed a correlation between elevated undernutrition scores in patients and factors such as older age, female gender, and marital status of being divorced or widowed. Furthermore, the findings suggest that an elevated risk score for undernutrition in patients is significantly associated with impaired Health-Related Quality of Life (HRQOL) compared to patients with normal nutrition.

## Acknowledgments

 We acknowledge the contributions of Tabriz University of Medical Sciences, Tabriz, Iran for providing facilities to the study.

## Competing Interests

 The authors declared no conflicts of interest.

## Ethical Approval

 Informed written consent was obtained from all participants. The study received ethical approval from the Ethics Committee of Tabriz University of Medical Sciences (NO: IR.TBZMED.REC.1396.816) (ID: 58437).

## Funding

 Tabriz University of Medical Sciencesprovides the facility to the study.
